# Construction of a multi-label odor prediction model based on molecular structures and olfactory receptor binding profiles with a novel interpretability framework

**DOI:** 10.1007/s44211-026-00900-6

**Published:** 2026-04-13

**Authors:** Yuta Wakutsu, Hiromasa Kaneko

**Affiliations:** Department of Applied Chemistry, School of Science and Technology, Meiji University, 1-1-1 Higashi-Mita, Tama-Ku, Kawasaki, Kanagawa 214-8571 Japan

**Keywords:** Odor prediction, Olfactory receptors, Receptor binding profiles, Multi-label classification, Positive likeness, Machine learning

## Abstract

Predicting odors from molecular structures is a long-standing challenge in chemoinformatics, especially in cases where structurally similar compounds, such as optical isomers, exhibit distinct odor perceptions. To address this, we developed a multi-stage odor prediction framework that integrates both molecular structures and olfactory receptor (OR) binding information. Recognizing that human olfaction is mediated by complex receptor–ligand interactions, we divided the process into three mechanistic stages: (1) prediction of molecular binding to ORs (classification), (2) estimation of binding strength (regression), and (3) prediction of odor presence based on receptor responses (classification). We further introduced a novel interpretability metric, Positive likeness, which estimates the contribution of specific receptors to the likelihood of each odor label. Using this framework, we demonstrated the ability to distinguish odor differences between optical isomers and to identify ORs that are potentially responsible for the perception of specific odor attributes. The model also enabled extrapolative odor prediction for molecules with unknown odor annotations, leveraging receptor information and label propagation. Our results highlight the importance of receptor-level descriptors in enhancing predictive performance and biological interpretability. This study provides a foundation for receptor-guided odor modeling and supports applications in fragrance design and sensory informatics.

**Graphical abstract:**

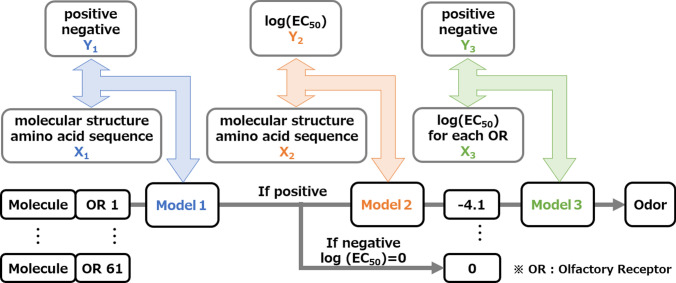

## Introduction

Flavors and fragrances are used in a variety of applications, including food, perfumes, cosmetics, and pharmaceuticals [1]. The global flavors and fragrances market size is projected to grow from $26.54 billion in 2022 to $36.49 billion in 2029, at a CAGR of 4.7%, with demand for new flavor molecules in the industry increasing every year [2]. Flavorings need to meet a variety of requirements in addition to having the desired odor characteristics for each particular application. In recent years, stricter safety and environmental regulations have made it difficult to use existing products in some cases, contributing to the growing demand for new flavor molecules [2], and thus, more efficient development of flavors and fragrances is required, however, the design and screening of conventional flavors and fragrances relies heavily on the experience of adepts, and is an iterative process of design, experimentation, and evaluation. The trial-and-error approach is not only costly in terms of significant financial and time costs and labor, but also would miss potential molecules with better properties [1, 2], and therefore, the use of machine learning has attracted much attention.

Quantitative structure–activity relationship (QSAR) [3] is the correlation between molecular structures and biological activity for molecules, and then, by replacing activity with odor attributes, QSAR models are constructed between odor attributes and molecular structures. However, predicting odor using QSAR models is difficult for the following reasons: (1) odorants change their odor significantly with slight structural or functional group changes; (2) odor perception is known to be subjective, and some researchers have argued that even trained experts may find it challenging to consistently communicate sensory experiences [4]. Although some team successfully constructed a model to predict odor attributes from molecular structures in an international crowdsourcing competition, the model has not been put to practical use due to the above difficulties [5]. To address the variability in sensory language, frameworks such as flavor wheels have been proposed [6], and recent natural language processing (NLP) approaches have explored mappings between mass spectral features and odor descriptors [7].

**Fig. 1:**
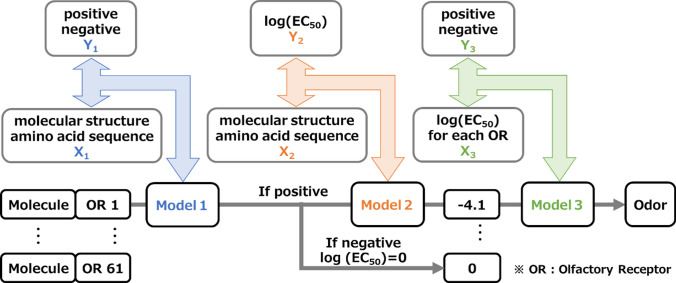
Odor prediction flow in our previous study [10]

Menthol differs in odor attributes due to slight structural differences. Specifically, *l*-Menthol has a significantly stronger minty odor than *d*-Menthol [8]. Regarding both odor attributes, Flavor-Base [9], a database on flavors, describes *l*-Menthol as "minty and cool" and *d*-Menthol as "less cool and musty woody than *l*-Menthol." To construct a model that discriminates odor differences between similar molecules, including optical isomers, it is necessary to extract the structural changes. In our previous study [10], we focused on the mechanism of human olfaction as an element other than structure that connects molecules and odorants. Humans have 388 types of olfactory receptors, each of which responds to odorants with different intensities, resulting in overall pattern recognition [11]. Olfactory perception is thought to arise from the brain’s interpretation of complex activity patterns across olfactory receptors, although the exact neural mechanisms remain an active area of research. For menthol, olfactory receptor MOR161-2 is activated only by *l*-Menthol, and MOR171-16 is activated only by *d*-Menthol [12]. In other words, if the response strength of each olfactory receptor is used as an indicator, it will be possible to represent almost all the odor attributes that humans perceive, which is expected to improve the accuracy of odor prediction models. In addition, the analysis of the constructed model can lead to the elucidation of the mechanism of smell from a biological point of view. Figure 1 shows a schematic diagram of our previously proposed method. We divided the olfactory mechanism into three steps. Specifically, we divided the odor prediction process into three steps: Step 1: prediction of binding to olfactory receptors, Step 2: prediction of binding activity, and Step 3: prediction of odor attributes from the combination of binding and binding strength. Therefore, three models corresponding to each step were constructed, i.e., Model 1, Model 2, and Model 3, respectively.

To validate the model, it was checked whether we could discriminate odor differences using molecules other than those used to construct the model. Our proposed method predicted odor differences between several similar molecules, including optical isomers. Although the prediction was successful for several molecules, the low prediction accuracy was an issue. Although the recent neural network model [13] has high prediction performance, interpretation of the model is difficult. Our proposed method uses information from olfactory receptors, which allows us to analyze the prediction results from a biological perspective. Our model can help to elucidate the relationship between molecules and odor attributes. By improving the prediction accuracy of our model, we can ensure the reliability of the interpretation of the relationship between molecules and odor attributes proposed by our model.

To improve the prediction accuracy of the model, it would be useful to examine novel protein descriptors and how to handle unknown odor attributes. Regarding the labeling of odor attributes, in general, when the odor attribute of a molecule is searched in a database, the odor attribute that the molecule possesses can be obtained. On the other hand, if an odor attribute is not listed, it is unclear whether the molecule does not have it or has not been investigated. Although our previous method labeled unknown odor attributes as negative, we need to consider a more appropriate way to handle them.

The objectives of this study are to construct an odor prediction model using novel protein descriptors, to interpret the relationship between molecules and odor attributes, and to construct an odor prediction model utilizing molecules with unknown odor attributes. The advantages of using highly accurate odor prediction models in flavor and fragrance development include: (1) reducing development time and cost by eliminating formulations that do not need to be tried and limiting the scope of the search, and (2) expanding the scope by suggesting new formulations [14]. Among machine learning research related to the human senses, compared to vision and hearing, including face recognition and automatic speech recognition [15, 16], examples of practical applications of machine learning for olfaction are scarce and research is lagging. This is not only a problem of machine learning, but the mechanism of olfaction itself is still in the developmental stage of elucidation, and the odor recognition mechanism of living organisms, including the relationship between molecules and odor attributes, has been increasingly studied in recent years, yet many aspects remain to be elucidated. This research will contribute to the development of research in the still developing field of olfaction by utilizing the interaction between molecules and olfactory receptors.

In this study, we used the difference in the number of known high-affinity versus low-affinity ligands for each receptor as an indicator of receptor selectivity. This approach is supported by prior studies that associate such ligand-binding asymmetry with differences in binding affinity, docking scores, or free energy estimates [17–21]. These studies suggest that selectivity can often be inferred from the differential distribution of ligand-binding profiles across receptors. Unlike our previous study [10], which used basic ligand-receptor binding indicators, this study introduces three-dimensional protein descriptors and proposes the Positive likeness metric for interpreting receptor-level contributions to odor perception.

Although several previous studies have attempted to predict odor categories or perceptual descriptors from molecular structures using traditional machine learning or deep learning methods, challenges remain in terms of interpretability, data sparsity, and handling multi-label odor associations. In particular, most existing approaches rely solely on labeled odor categories and overlook the potential role of olfactory receptor interactions in odor perception. This study aims to address these issues by integrating odor labels and receptor-binding data into a unified predictive model, while also introducing a novel interpretability index, Positive likeness, that can deal with unknown or missing labels in a semi-supervised manner.

## Method

### Dataset

The data set used in this study is collected from the reference [22]. The data show the log(EC50) of 63 molecules for 61 different olfactory receptors, and the olfactory receptors include both mouse and human receptors. The log(EC50) is the logarithm of the half maximal effective concentration (EC50) [23], which is the concentration at which a drug or antibody produces 50% of its maximal response. In this study, it represents the concentration of a molecule in the reaction in which the molecule binds to the olfactory receptor. From this data set, we prepared data sets for three models. Molecular odor attributes are retrieved from the database OlfactionBase [24]. The data set is split 8:2 between training and test data, respectively, and the test data is used to validate the models.

The data set for Model 1 contains 3843 samples, all of which are combinations of molecules and olfactory receptors. log(EC50) = 0 samples are considered negative and log(EC50) > 0 samples are considered positive. The data set for Model 2 includes only those samples that were considered positive in the data set for Model 1. The objective variable is log(EC50) and the number of samples is 333. In preparing the data set for Model 3, molecules are obtained from OlfactionBase and the literature data used in Models 1 and 2. Because there exists no data for odor attributes in the literature data, it is obtained from the database. In total, log(EC50) for 61 different olfactory receptors for a total of 2883 molecules and the odor data they possess are obtained.

### Descriptors

Molecular descriptors include Morgan fingerprints (Morgan) [26], RDKit fingerprints (RDKit) [27], and MACCS Keys fingerprints (MACCS Keys) [28], which are calculated with RDKit software [25], and three types of protein descriptors are considered. The first one is a descriptor set of amino acid sequence information (protr) computed using the protr package [29]. The protr package is a tool for feature extraction of protein sequences available in Python, including amino acid composition and autocorrelation. The second one is a descriptor set selected with Boruta [30] from a three-dimensional descriptor set [31] (3D + Boruta). For each atom in the protein, a list of amino acids in a three-dimensional space with a radius of 5.0 Å is prepared, storing 1 if the atom has that structure and 0 otherwise. The third one is a latent variable (ESM-2) in the protein language model ESM-2 [32], with which there exist examples of its contribution to improving the predictive performance of models [33, 34]. In this study, we use protloc-mex-x [35, 36], a Python library for feature extraction using ESM-2. For the input array, four latent variables are obtained from the last intermediate layer of ESM-2. The name, properties and dimensionality of each variable are given as follows:

CLS: Descriptor set in which the meaning of the entire text is stored. Information on the potential 3D structure of the amino acid sequence. Often used as input for classification models (1280 dimensions).

EOS: Descriptor set used as input to the classification model although less frequent than CLS (1280 dimensions).

Mean: Descriptor set averaged over 1280 dimensions of latent variables for each amino acid by dimension (1280 dimensions).

Segments: Descriptor set with array divided into 10 groups and Mean calculated for each group (12,800 dimensions).

The Boruta algorithm was applied and we evaluated three different percentile thresholds for selecting features (p = 80, 90, and 100) and adopted the subset that resulted in the highest prediction accuracy. This selection strategy ensured the retention of informative variables while mitigating overfitting.

### Modeling methods

The classification methods considered in Models 1 and 3 are logistic regression analysis [37], linear discriminant analysis [38], naive Bayes classifier (NB), k-nearest neighbor algorithm, linear support vector machine [39], non-linear support vector machine [39, 40], decision tree (DT) [41], random forest (RF) [42], light gradient boosting machine (LGBM) [43], eXtreme gradient boosting (XGB) [44], gradient boosting decision tree (GBDT) [41]. The regression analysis methods considered in Model 2 are partial least squares regression [45], ridge regression [46], least absolute shrinkage and selection operator [47], elastic net [48], linear support vector regression [49], non-linear support vector regression (NLSVR) [49], RF, LGBM, XGB, DT, GBDT, gaussian process regression (GPR) [50].

Classification methods often fail to discriminate between molecules that bind to each other and olfactory receptors, resulting in more negative samples than positive samples. Therefore, in Models 1 and 3, the clustering was conducted with an undersampling method [51] for only training data to reduce the number of majority samples and keep the number of samples balanced. The clustering method used k-means +  + [52] in this study. In an unbalanced data set with *N* minority samples and *M* majority samples, the majority sample is divided into *k* clusters. *N/k* samples are then randomly extracted from each cluster. *m*_*i*_ (i = 1,2…*k*) is the number of samples to be extracted from the cluster. *M*_new_ is the total number of samples to be extracted and its ratio to *N* is close to 1.

We set the cluster number *k* = 3 to ensure that the number of samples extracted from each cluster did not exceed the actual number of samples within that cluster. The number of extracted samples per cluster was calculated as:

1 $${\mathrm{Extracted}} {\mathrm{samples}} {\mathrm{per}} {\text{cluster }} = {\text{ Number}} {\mathrm{of}} {\mathrm{minority}} {\mathrm{samples}}/k$$

and *k* = 3 was found to satisfy this condition without resulting in overly small clusters.

### Evaluation indexes

The evaluation indexes for Models 1 and 3, which are classifiers are accuracy, precision, recall, and F-value, which are calculated as follows:

2 $$\begin{array}{*{20}c} {Accuracy = \frac{TP + TN}{{TP + FP + FN + TN}}} \\ \end{array}$$

3 $$\begin{array}{*{20}c} {Precision = \frac{TP}{{TP + FP}}} \\ \end{array}$$

4 $$\begin{array}{*{20}c} {Recall = \frac{TP}{{TP + FN}}} \\ \end{array}$$

5 $$\begin{array}{*{20}c} {F - value = \frac{2 \times Precision \times Recall}{{Precision + Recall}}} \\ \end{array}$$

where *TP* is the number of true positives, *TN* is the number of true negatives, *FP* is the number of false positives, and *FN* is the number of false negatives. The closer the indexes are to 1, the better the performance of classification models.

The evaluation indexes in Model 2, which is regression model, are coefficient of determination *r*^2^, mean absolute error (MAE), and root mean squared error (RMSE), which are calculated as follows:

6 $$\begin{array}{*{20}c} {r^{2} = 1 - \frac{{\mathop \sum \nolimits_{i = 1}^{n} \left( {y^{\left( i \right)} - y_{EST}^{\left( i \right)} } \right)^{2} }}{{\mathop \sum \nolimits_{i = 1}^{n} \left( {y^{\left( i \right)} - y_{mean} } \right)^{2} }}} \\ \end{array}$$

7 $$\begin{array}{*{20}c} {MAE = \frac{{\mathop \sum \nolimits_{i = 1}^{n} \left| {y^{\left( i \right)} - y_{EST}^{\left( i \right)} } \right|}}{n}} \\ \end{array}$$

8 $$\begin{array}{*{20}c} {RMSE = \sqrt {\frac{{\mathop \sum \nolimits_{i = 1}^{n} \left( {y^{\left( i \right)} - y_{EST}^{\left( i \right)} } \right)^{2} }}{n}} } \\ \end{array}$$

where *y*^(*i*)^ is the value of the objective variable for the i*-th* sample, *y*_EST_^(*i*)^ is the estimated value of the objective variable for the *i-th* sample, *y*_mean_ is the mean value of the objective variable, and *n* is the number of samples.

### Interpretation of relationships between molecules and odor attributes

The target odor attributes are those with high prediction accuracy in Model 3. The variable importance is calculated for the constructed Model 3. Here, the variable importance of Model 3 indicates the magnitude of the contribution of log(EC50) of each receptor to the prediction of odorants. In addition, a quantitative evaluation of whether the molecule should or should not bind to the olfactory receptor is performed. We use the predictions of the presence or absence of binding between the molecule and the olfactory receptor in Model 1. The absolute difference in the percentage of molecules that bind to olfactory receptors between molecules with and without the odor attribute of interest is calculated.

### Handling unknown odor attributes

When we search the odor database for a molecule, we obtain the odor attribute that the molecule has as defined by previous studies. Although these data are useful for research in the field of olfaction, when considering an odor attribute that a molecule does not have, the absence of a certain odor attribute in the database does not mean that the molecule does not have that odor attribute. Sensory evaluation does not exhaustively confirm the presence or absence of an odor attribute listed in the database. In this study, such samples of unknown molecule/odor attribute combinations are referred to as Unknown samples.

We examined two ways to handle Unknown samples: the first one is the conventional method of labeling Unknown samples as negative; the second one is to utilize Unknown samples as they are: the likelihood that an Unknown sample is positive is expressed as a continuous score between 0 and 1. For this proposal, we focused on the quantitative estimate of QED [53], which is a quantitative measure between 0 and 1 of drug-likeness of each compound, including molecular weight, logP, number of hydrogen bond donors, number of hydrogen bond acceptors, polar surface area, number of rotatable bonds, and aromatic ring. It was modeled using a dataset of 771 oral drugs, and the weighting of each descriptor was determined to maximize Shannon’s entropy. The fact that inactive molecules are not used in the modeling is highly compatible with the challenge of labeling odor attributes. Based on this idea, we do not use negative labels in this study, but introduce an index: Positive likeness that expresses the likelihood that a molecule has the predicted odor attribute as a continuous score from 0 to 1. Although Model 3 is classification models, where the objective variable is positive and negative for an odor attribute, not negative samples but Unknown samples exist in the odor database. In this study, by using positive samples and Unknow samples, Positive likeness is calculated.

**Fig. 2:**
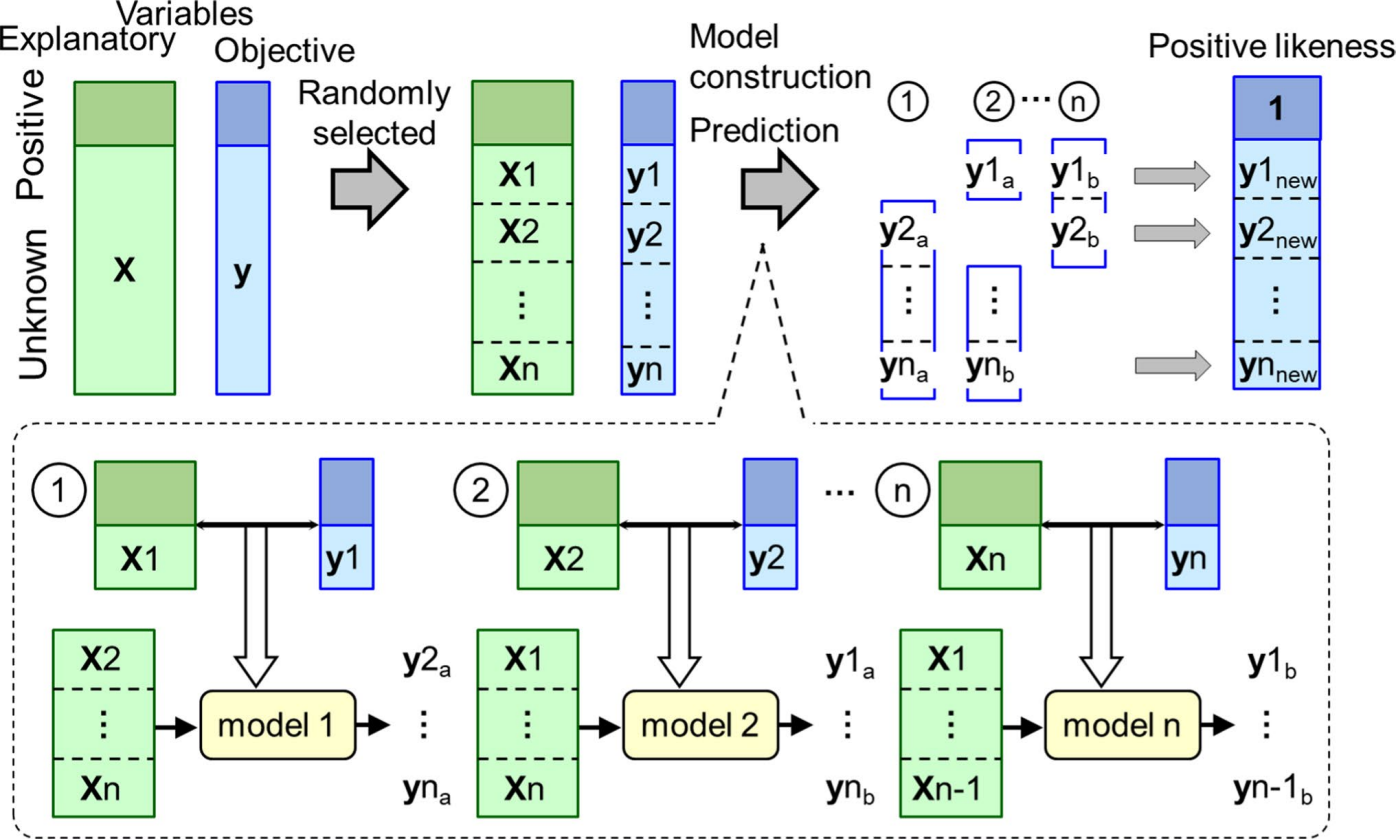
Basic concept of calculation of the proposed positive likeness based on random sampling of Unknown samples and multi model construction

The basic concept of calculation of Positive likeness is shown in Fig. 2. First, the data set is divided into positive samples and Unknown samples. Next, Unknown samples are randomly selected as negative samples and merged with the positive samples, and a classification model is constructed using the merged samples. Then, the Unknown samples, which were not selected, are input into the model, and positive or negative are predicted. The total number of randomly selected Unknown samples is set equal to the total number of positive samples. After repeating the above process from random selection to prediction *n* times, the number of times that each Unknown sample is predicted to be positive is divided by the total number of times, and a value between 0 and 1 is provided. In this study, the measure provided with the proposed method is called “Positive likeness”. Among the Unknown samples, Positive likeness is higher for samples that have the same characteristics as the positive samples.

## Results and discussion

### Model 1

Morgan, RDKit, and MACCS Keys were used to construct machine learning models and compare their predictive accuracy. Protr was used as the protein descriptor set. The evaluation indexes of the top six most predictive models in test data are shown in Table 1. 61 olfactory receptors used in this study were considered to have specific structures favorable for binding among the 166 sub-structures employed by MACCS Keys. Because some of the substructures, such as aromatic rings and five-membered rings, are difficult to interpret with radius = 2 in Morgan fingerprints, it is considered more effective to judge whether or not the olfactory receptor binds to a large substructure than to a local substructure.

**Table 1:** Evaluation indexes of the top six most predictive models in test data for classifying the presence or absence of binding to olfactory receptors for Model 1

Method	Molecular descriptor	Accuracy	Precision	Recall	F-value
XGB	MACCS Keys	0.880	0.861	0.912	0.886
LGBM	MACCS Keys	0.865	0.829	0.926	0.875
XGB	RDKit	0.842	0.831	0.868	0.849
LGBM	Morgan	0.842	0.822	0.882	0.851
LGBM	RDKit	0.835	0.829	0.853	0.841
XGB	Morgan	0.812	0.803	0.838	0.820

### Model 2

**Fig. 3:**
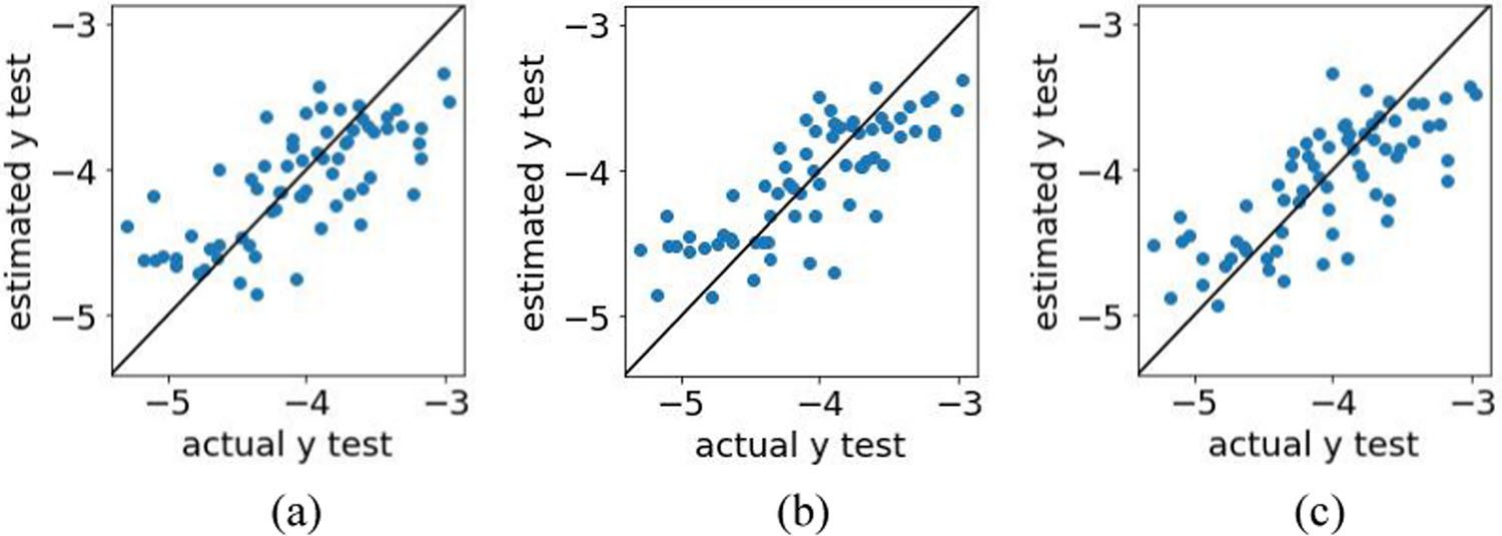
Plots of measured vs. estimated plots for model 2 for each of the three protein descriptors for Model 2. **a** protr, **b** 3D + Boruta (**c**) ESM-2(EOS)

Machine learning models were constructed using protr, 3D + Boruta, and ESM-2 and their predictive accuracy was compared. For the molecular descriptors, we used the best combination of Morgan, RDKit, and MACCS Keys that had the highest predictive accuracy. Table 2 shows the evaluation indexes in test data for the best combination of regression method and molecular descriptor set for each protein descriptor, and Fig. 3 shows scatter plots between actual values vs. estimated values with the best method. 3D + Boruta and ESM-2 with 3D structure exceeded protr with only amino acid sequence information in all three evaluation indexes. In Fig. 3, the samples with the three-dimensional structure are located near the diagonal line from the highest to the lowest values of log(EC50). The importance of protein steric structure in the activity of molecules and olfactory receptors was demonstrated.

**Table 2:** Evaluation indexes in test data for regression analysis of log(EC50) of binding to olfactory receptors for Model 2

Protein descriptor	Molecular descriptor	Method	*r*^2^	RMSE	MAE
protr	Morgan	RF	0.514	0.392	0.310
3D + Boruta	Morgan	GBDT	0.613	0.350	0.291
ESM-2(EOS)	Morgan	LGBM	0.569	0.369	0.297

The difference in the best-performing molecular descriptors between Model 1 and Model 2 may reflect the different structural information required by the two tasks. Model 1 classifies whether binding occurs or not, and this decision can depend mainly on the presence or absence of relatively coarse and characteristic substructures, which are well represented by fixed structural keys such as MACCS Keys. In contrast, Model 2 predicts the magnitude of binding strength for molecule–receptor pairs that were already classified as binders, and therefore may require finer information on local atomic environments that influence interaction strength. Morgan fingerprints can better capture such local structural variations. In addition, the nonlinear relationship between local structural differences and log(EC50) can have been effectively modeled by GBDT. We note that this interpretation is a mechanistic hypothesis based on the observed descriptor performances and the nature of the two prediction tasks, and was not independently validated in the present study.

### Model 3

In this study, we focused on citrus and musk odor attributes and collected odorants based on 106 “Primary Odors” in OlfactionBase. Citrus odor attribute is considered one of the most important fragrances in the fragrance industry and are used in a wide range of applications, and thus, there is a constant need for new fragrances that meet the criteria required for specific applications [54]. A search of OlfactionBase revealed three citrus-related odor attributes: "Lemon," "Citrus," and "Terpenes/Pine/Lemon" (for simplicity, we use “Terpenes”) were obtained. Furthermore, in addition to the citrus odor attribute, additional flavors can be required for some purposes. Therefore, we considered the odor attribute to be predicted in combination with citrus and found many references to “Fresh”, “Fruity”, "Sharp/Pungent" (for simplicity, we use “Sharp”), “Sweet”, and “Waxy”, which we obtained from OlfactionBase.

Next, we focused on Musk as an odor attribute in high demand other than Citrus. While natural musk has a sweet and complex aroma among the many fragrance ingredients and has been widely used as a high-end fragrance for many years, there are ethical issues because natural musk is obtained from the gonadal sacs of musk deer, and comparisons of musk deer are currently prohibited under the Washington Convention, making it extremely difficult to obtain natural musk [55]. This has led to the development of synthetic fragrances, but stricter environmental regulations and the increasing demand for musk fragrances themselves have made it desirable to develop new musk-based fragrances. Thus, musk was chosen as a non-citrus odor attribute, and a search in OlfactionBase yielded an odor attribute called “Ambrosial” with only “Musk” in the "Sub-Odors". The results of the search in OlfactionBase yielded the odor attribute "Ambrosial”. In this study, we call it “Musk” for the sake of clarity. As a result of searching for molecules with the above 9 odor attributes, 2820 molecules were obtained. We constructed a total of 9 models to predict the presence or absence of these odor attributes.

Model 3 was constructed based on the predictions of Model 2, which was constructed using each of the three protein descriptor sets considered in Model 2. Table 3 shows the evaluation indexes in test data for the classification results in each model. The highest F-value was 0.970 for Musk, and all but one negative sample was predicted correctly.

**Table 3:** Evaluation indexes in test data for Model 3

Odor attribute	Protein descriptor	Method	Accuracy	Precision	Recall	F-value
Lemon	protr	XGB	0.752	0.788	0.905	0.842
Citrus	ESM-2	XGB	0.718	0.737	0.760	0.749
Terpenes	protr	RF	0.594	0.606	0.664	0.634
Fresh	ESM-2	RF	0.632	0.603	0.577	0.590
Fruity	ESM-2	XGB	0.663	0.686	0.680	0.683
Sharp	3D + Boruta	XGB	0.651	0.652	0.600	0.625
Sweet	3D + Boruta	GBDT	0.769	0.781	0.968	0.864
Waxy	protr	NB	0.764	0.744	0.792	0.767
Musk	ESM-2	DT	0.968	0.944	1.000	0.971

### Comparison with conventional methods

**Fig. 4:**
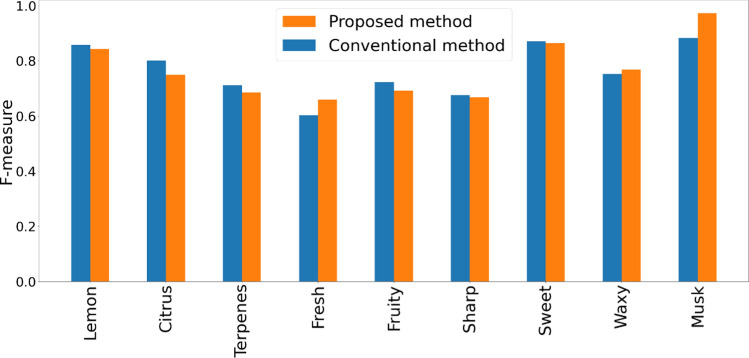
F-measure for the proposed and conventional methods in Model 3

The proposed method utilized information from olfactory receptors, whereas the conventional method constructed a model that predicted odor attribute from only molecular structures. Molecular descriptors were optimized for each of the nine odor attributes, which had relatively large numbers of samples in the OlfactionBase database, from the three sets of MACCS Keys, Morgan, and RDKit. For protein descriptors, we used protr. The data set was the same as in Model 3, and machine learning model was constructed for each odor attribute. The number of samples was 2883 for all the odor attributes, the objective variable was the presence or absence of the odor attribute of interest for each model, and the explanatory variables were the actual or predicted log(EC50) values for the 61 odorant receptors. Figure 4 compares the F values for the proposed and conventional methods [10], and Table 4 shows the evaluation indexes of the method with the highest percentage of correct answers for each odor attribute. From Fig. 4, the proposed method outperformed the conventional method in terms of Fresh, Waxy, and Musk. In particular, comparing Tables 4 and 5 for Musk, the F value improved from 0.882 to 0.970. Thus, we focused on the structure of the molecule that possesses the musk odor attribute. Musk can be divided into four groups: Nitro Musk, Polycyclic Musk, Macrocyclic Musk, and Alicyclic Musk [56]. Each group shares common structural features, but there is little similarity between groups. In such a case, a model that predicts an odor attribute only from chemical structures would not be able to learn the relationship between the presence or absence of odor attributes and the common substructure. In the proposed method, although Models 1 and 2 use molecular structures as input, the input for the final Model 3 is log(EC50) for olfactory receptors. This allows consideration of not only molecular structures but also the binding patterns of 61 types of olfactory receptors, enabling accurate prediction even when common substructures differ. Therefore, the biological approach would be effective for some odor attributes, and although Musk can be predicted with high accuracy based on structure alone compared to other odor attributes, it is important to improve the accuracy of the model using the proposed method to ensure the reliability of the hypothesis proposed by this model in the interpretation of the relationship between molecules and odor attributes.

**Table 4:** Evaluation indexes for predicting odor attributes for Model 3

Odor attribute	Descriptor	Method	Accuracy	Precision	Recall	F-measure
Lemon	Morgan	GBDT	0.763	0.765	0.974	0.857
Citrus	RDKit	RF	0.793	0.857	0.750	0.800
Terpenes	RDKit	RF	0.646	0.675	0.751	0.711
Fresh	RDKit	XGB	0.626	0.587	0.620	0.603
Fruity	RDKit	GBDT	0.706	0.696	0.753	0.723
Sharp	Morgan	XGB	0.694	0.695	0.656	0.675
Sweet	RDKit	NSVM	0.780	0.788	0.970	0.870
Waxy	RDKit	RF	0.752	0.738	0.766	0.752
Musk	Morgan	RF	0.871	0.882	0.882	0.882

**Table 5:** Examples of molecules with high positive likeness

Odor attribute	Molecule	Predicted odor attribute	Positive likeness
Lemon Terpenes	2-Heptenoic acid	Citrus	0.956
	2-Methylpent-2-enal		0.951
	Isotridecane		0.875
	p-Mentha-1,3,8-triene		0.800
Herbaceous	Dihydrobovolide	Minty	1.00
	2-Methoxypyridine		1.00
Fishy/ Rancid	Putrescine	Ammonia	0.978

A strict end-to-end validation of the full three-stage pipeline remains difficult at present because a sufficiently large dataset containing both experimentally measured olfactory receptor responses and comprehensive odor annotations for the same molecules is not available. Therefore, in this study, each stage was evaluated independently using held-out test data, and the significance of the overall framework was assessed indirectly through downstream evidence. Specifically, the proposed framework improved the predictive performance over the conventional structure-only approach for several odor attributes, successfully discriminated odor differences between optical isomers, and enabled receptor-level interpretation for the Musk odor attribute. Although these results do not constitute complete external validation of the entire pipeline, they support the practical value of incorporating receptor-level information for both prediction and interpretation.

### Identification of optical isomers

**Fig. 5:**
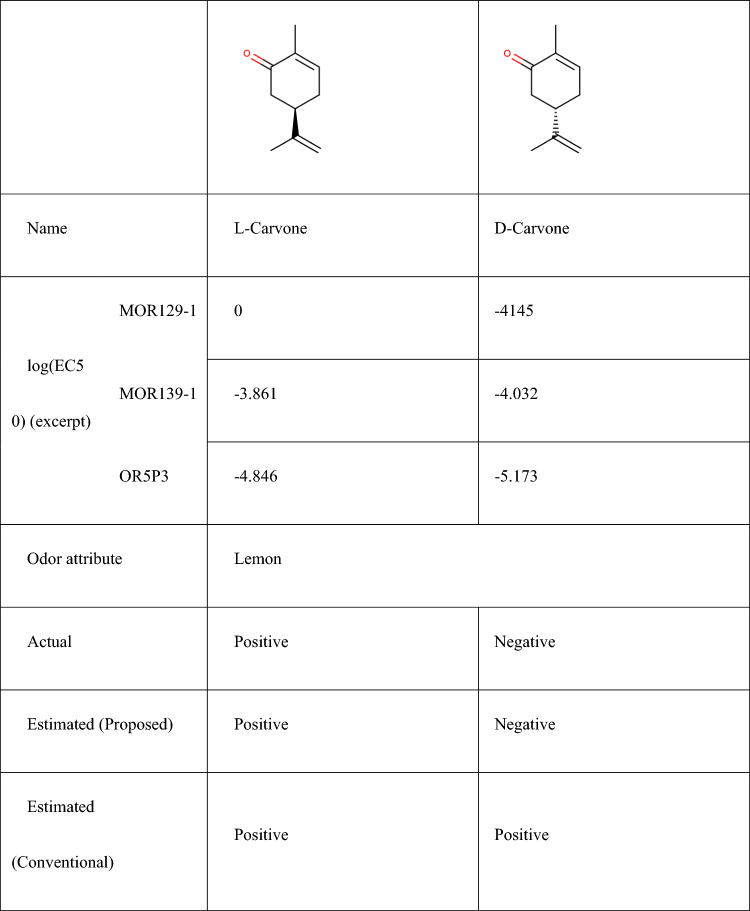
Examples of predicted optical isomers

The optical isomers were extracted from the data set used in Model 3, which was reconstructed using the remaining molecules to predict the odor attribute of the optical isomers. Figure 5 shows the optical isomers whose odor differences were discriminated with the proposed model. For example, the difference in odor attribute between *l*-Carvone and *d*-Carvone was identified, and the log(EC50) values input into Model 3 were different between the two isomers.

### Interpretation of relationships between molecules and odor attributes

**Fig. 6:**
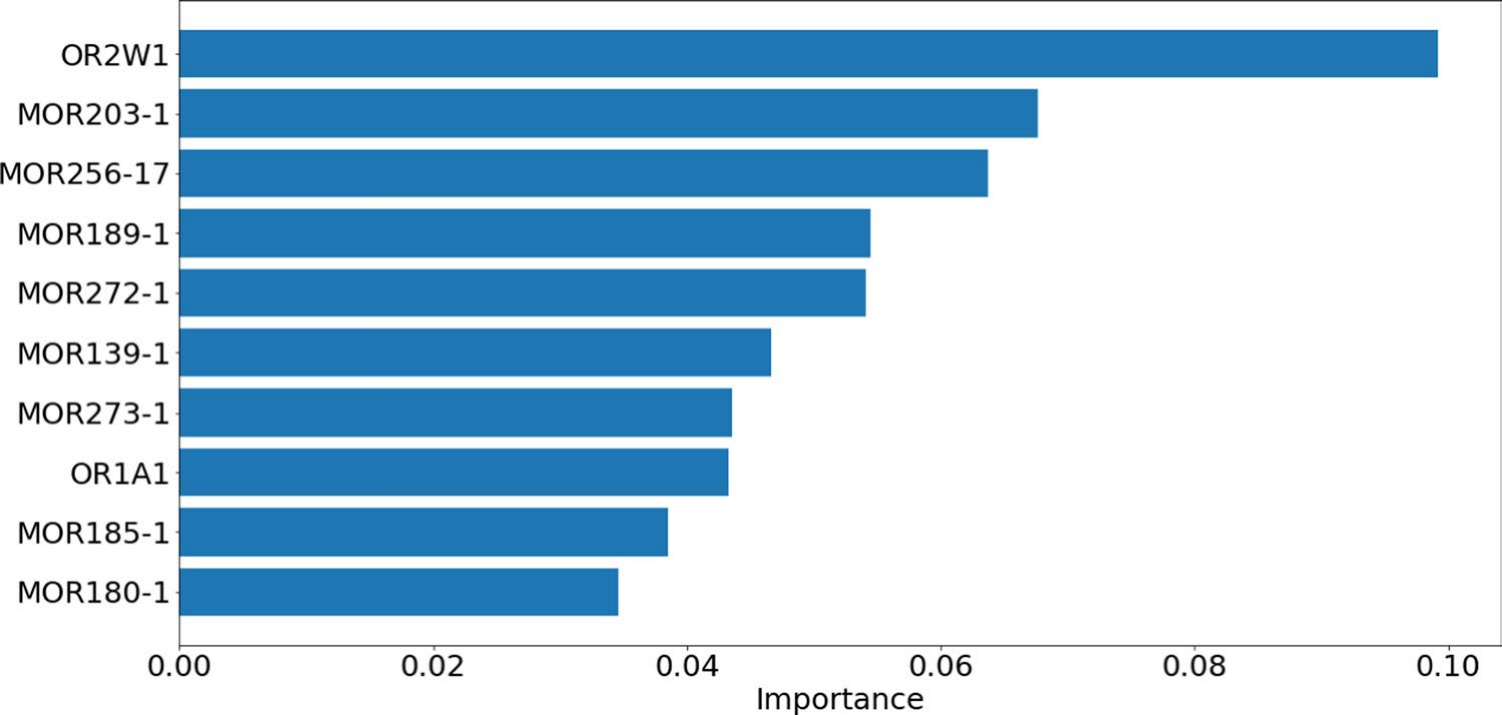
Top 10 olfactory receptors important for predicting Musk for Model 3

Musk, which had the highest F value, was focused on in this study. First, the variable importance of Model 3 of Musk was calculated. Figure 6 shows the top 10 olfactory receptors in order of importance, and OR2W1 was the most important olfactory receptor in predicting Musk.

We conducted a quantitative evaluation of whether molecules should bind to olfactory receptors or not. The absolute difference in the percentage of molecules that bind to olfactory receptors between those with and without Musk was calculated. For example, in the case of MOR233-1, there exist a total of 83 Musk molecules, 42 of which bind to MOR233-1. In this case, the ratio of Musk molecules binding to MOR233-1 is 42/83 (0.51). On the other hand, there are 2827 non-Musk molecules in total, of which 596 bind to MOR233-1. In this case, the binding ratio of non-Musk molecules to MOR233-1 is 596/2827 (0.211). The absolute difference between the binding ratios of Musk and non-Musk molecules to MOR233-1 is 0.51−0.211 = 0.30. If the absolute difference is positive, it can be interpreted that the corresponding olfactory receptor is a receptor whose binding is essential because the molecule has Musk, and if the absolute difference is negative, it can be interpreted that the receptor should be avoided because the molecule has Musk.

**Fig. 7:**
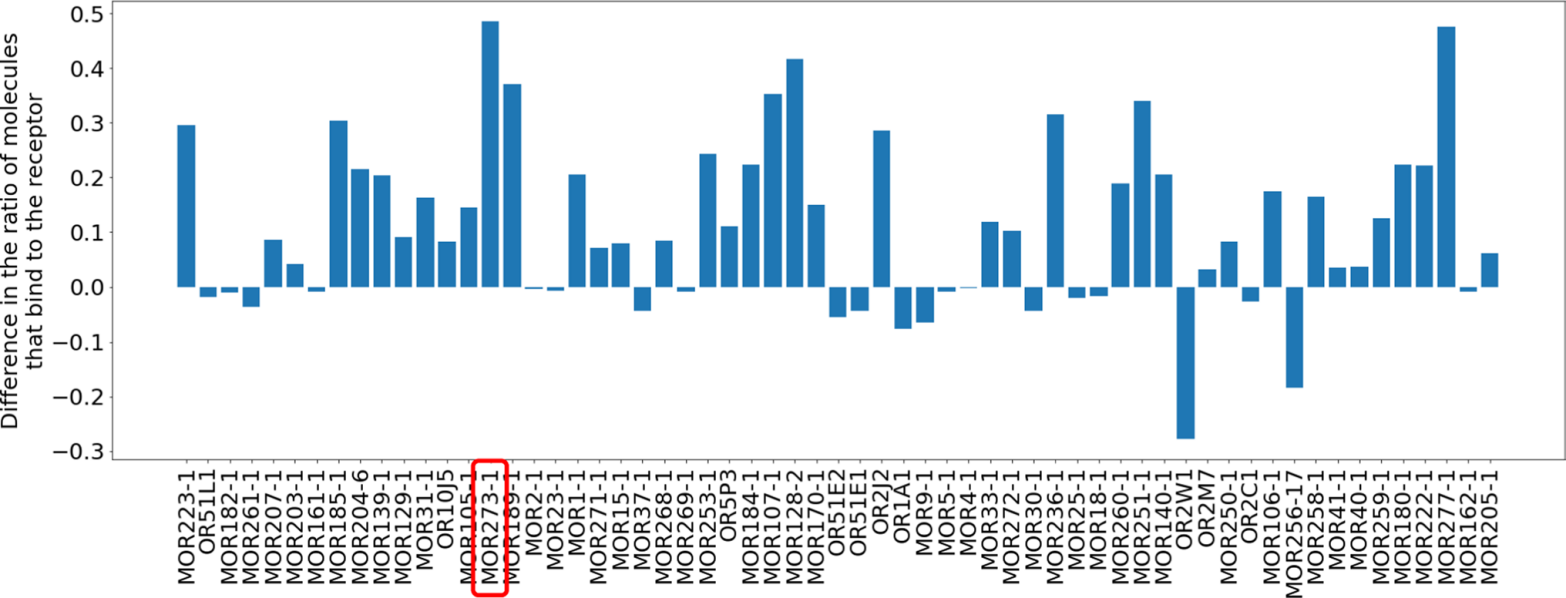
Difference in the percentage of molecules that bind to each olfactory receptor between Musk and non-Musk molecules

Figure 7 shows a bar graph of the absolute difference in the binding ratio of Musk and non-Musk molecules to 61 different olfactory receptors. MOR273-1 and MOR277-1 (circled in red) were identified as olfactory receptors with a positive absolute difference in binding ratio, and OR2W1 (circled in orange) was identified as an olfactory receptor with a negative absolute difference. Thus, MOR273-1 and MOR277-1 are receptors that must bind to the molecule in order for it to possess Musk, while OR2W1 is a receptor that must be avoided in order for the molecule to possess Musk. In particular, OR2W1 is consistent with the consideration of variable importance in Model 3, reinforcing the reliability of the present proposal.

### Utilization of combinations of unknown molecules and odor attributes

In this study, after searching for molecules in OlfactionBase to obtain odor attributes, the nine target odor attributes were labeled positive if they had them and negative if they did not. In other words, the negative samples used in Model 3 are molecules that were not listed in OlfactionBase and are strictly Unknown samples that can be positive. Therefore, the Positive likeness was calculated for these samples in this study.

Odor perception is inherently multi-label, and individual compounds are often associated with multiple, diverse sensory terms. We addressed this by modeling the problem as a classification task, not regression. Specifically, the Positive likeness score is derived from the output probabilities of a classifier that independently estimates the presence or absence of each odor descriptor for a given molecule. Thus, the output is a continuous-valued likelihood for each label, rather than a single regression target or mutually exclusive classification.

For the data, out of the 105 odor attributes in OlfactionBase, molecules for which no structural information was available were excluded, and odor attributes with 10 or more molecules having that odor attribute were targeted. As a result, 92 odor attributes and 3887 molecules were obtained. The total number of samples is 357,604 (92 × 3887), which indicates all combinations of odor attributes and molecules. 40,012 were positive samples and 317,592 were non-positive samples, which were handled as negative samples of Unknown samples.

First, we verified the effectiveness of the Positive likeness calculation method using virtual samples. The number of positive samples and the number of negative samples were set equal to the odor data set. We set two explanatory variables, × 1 and × 2, and changed the settings of the values of × 1 and × 2 so that the positive samples and the unknown samples would have different distributions. Specifically, positive samples are set to random numbers between 0 and 1 for two variables, unknown samples are set to random numbers between − 1 and 1, and when expressed in coordinates, positive samples are set to be distributed in the first quadrant and unknown samples are set to be distributed in all quadrants.

**Fig. 8:**
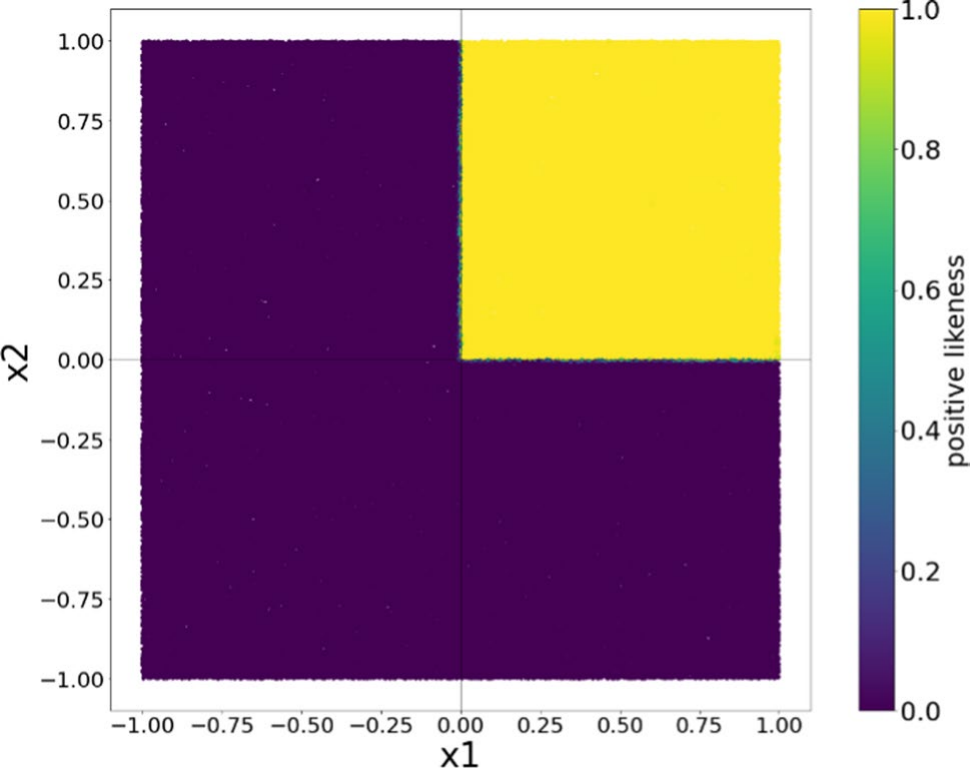
Demonstration of Positive likeness in two x-variables for virtual unknown samples

Based on the generated virtual data set, the proposed method for calculating the Positive likeness of unknown samples was verified. Since the number of samples is very large, LGBM was used as the model building method from the viewpoint of computational cost and expected prediction accuracy. Figure 8 shows a plot of the coordinates of the predicted virtual unknown samples, colored according to Positive likeness. The Positive likeness of the unknown samples distributed in the first quadrant was predicted to be high, while the other unknown samples were predicted to be low. It was confirmed that the proposed Positive likeness calculation method is effective to classify Unknown samples.

Table 5 shows examples of molecules that were predicted to be high Positive likeness for odor attributes similar to the one they possess. Some of the molecules with citrus odor attribute, such as Lemon and Terpenes, were predicted to have a high Positive likeness of also possessing Citrus. Next, some of the molecules with the herbal Herbaceous odor attribute were predicted to have a high probability of also having Minty. Finally, some of the Fishy/Rancid molecules were predicted to have a high probability of also possessing Ammonia. Thus, by calculating the Positive likeness of the odorants that were not present, we could identify the odorants that could potentially be present.

## Conclusion

In this study, we extended our previous odor prediction model [10] by introducing novel protein descriptors that incorporate the three-dimensional structural features of olfactory receptors. This allowed us to more accurately model the relationship between molecular structure and perceived odor attribute via receptor-level interactions, and to address the challenge of predicting unknown or unlabeled odor attributes. Using the new receptor descriptors, we observed improved predictive performance in three out of nine odor attributes. In particular, for the Musk odor attribute—which showed high prediction accuracy—we identified key receptor interactions contributing to its perception by analyzing the variable importance scores and the absolute differences in receptor-binding proportions between molecules with and without Musk labels. These findings enabled us to propose receptor-related conditions necessary for a molecule to exhibit Musk-like odor attribute. Our results suggest that simultaneous advancement of both prediction accuracy and interpretability is essential, and that model-derived interpretations grounded in high-performing predictions offer more reliable insights into odor mechanisms.

For molecules that do not have odor annotations in the database, which are conventionally treated as negative samples, we instead proposed an index called Positive likeness to represent the potential presence of the odor attribute. After verifying Positive likeness using virtual numerical simulation data, we used actual compounds to confirm that compounds with high Positive likeness for a given odor attribute actually possess that odor attribute. We plan to set threshold values for each odor attribute and to reconsider the target odor attributes. The proposed method can be one of the useful methods in dealing with the phenomenon of subjective and ambiguous definition of odor attributes.

While the present study demonstrated variable importance analysis using Musk as an example, the proposed framework can be applied to other odor descriptors as well. However, directly linking subtle molecular structural changes to receptor responses remains a challenging task due to the complexity of olfactory mechanisms. Future work will address this by extending interpretability analysis to multiple odor attributes and developing approaches to qualitatively correlate molecular modifications with receptor activity patterns. Moreover, the Positive likeness approach proposed in this study, while effective for handling unknown odor attributes, remains heuristic in nature. Incorporating semi-supervised learning strategies such as PU-learning or confidence propagation will be explored to enhance its theoretical rigor and reliability. In addition, relatively low predictive performance observed for certain odor attributes, such as Terpenes and Fresh, may be attributed to limited sample sizes and inconsistencies in labeling. We aim to improve data curation and labeling quality in future datasets. Furthermore, while the number of clusters k = 3 was heuristically chosen in clustering-based undersampling to avoid overly small clusters, the optimization of k through data-driven approaches will also be considered in future research. We also plan to expand the molecular and receptor datasets by incorporating additional odor-related annotations from public resources such as FlavorDB and PubChem, which is expected to enhance the robustness and predictive performance of the proposed models. Finally, although this study focused on in silico predictions, future work will include experimental synthesis and validation of model-suggested molecules, particularly for musk-like compounds, to enhance practical applicability and industrial relevance. Additionally, to support real-world use by non-expert users in fragrance design, we aim to develop a user-friendly web-based visualization tool that displays receptor–odor relationships and Positive likeness scores to facilitate compound screening and interpretation.

Furthermore, while this study utilized odor annotations from OlfactionBase, including “Primary Odors” and "Sub-odors," it is important to recognize that sensory descriptors are influenced by cognitive, linguistic, and cultural variability. To improve the generalizability and robustness of our findings, future studies should consider validating the models using alternative datasets curated from clearly defined primary sources in the Pyrfume project, or GoodScents. Such cross-dataset validation would help assess the reproducibility of odor prediction across different sensory vocabularies and historical contexts. Future work should focus on full end-to-end validation using datasets in which receptor activity measurements and odor annotations are jointly available for the same molecules.

Regarding the interpretation of musk-related receptor activation, we acknowledge that our analysis of the activation ratio and feature importance is based on a specific dataset and modeling framework, and does not comprehensively capture the complex biology of musk perception. Previous studies have identified several human olfactory receptors (e.g., OR5AN1, OR5A2, OR1A1, OR4D6) involved in musk detection, and different structural classes of musk odorants may activate different receptors. Furthermore, our dataset included both human and mouse olfactory receptors, which limits the direct extrapolation of our results to human perception. In future work, we plan to incorporate musk-specific datasets and focus on human ORs to validate and refine these findings.

In addition, we plan to incorporate additional datasets that use standardized odor lexicons [57–59], which provide explicit negative labels for odor descriptors. These resources could serve both as alternative training datasets for Model 3 and as independent benchmarks for validating the Positive likeness score. By integrating such standardized datasets, we aim to further improve the generalizability and interpretability of our odor prediction framework.

We hope that the results of our research will promote the development of odorants.

## Data Availability

Data supporting the findings of this study are available in [25, 36]. We used scikit-learn https://scikit-learn.org/stable/ to construct machine learning models, and RDKit https://www.rdkit.org/ to handle chemical structures and calculate molecular descriptors.
